# Re(I) Complexes as Backbone Substituents and Cross-Linking Agents for Hybrid Luminescent Polysiloxanes and Silicone Rubbers

**DOI:** 10.3390/molecules26226866

**Published:** 2021-11-14

**Authors:** Egor M. Baranovskii, Victoria V. Khistiaeva, Konstantin V. Deriabin, Stanislav K. Petrovskii, Igor O. Koshevoy, Ilya E. Kolesnikov, Elena V. Grachova, Regina M. Islamova

**Affiliations:** 1 Institute of Chemistry, St Petersburg University, 198504 St. Petersburg, Russia; baranovskij.1985@mail.ru (E.M.B.); st034645@student.spbu.ru (V.V.K.); deriabin.k@yahoo.com (K.V.D.); s.petrovsky@spbu.ru (S.K.P.); 2 Department of Chemistry, University of Eastern Finland, 80101 Joensuu, Finland; igor.koshevoy@uef.fi; 3 Center for Optical and Laser Materials Research, Research Park of St Petersburg University, 198504 St. Petersburg, Russia; ilya.kolesnikov@spbu.ru

**Keywords:** functional polysiloxanes, rhenium complexes, luminescent polysiloxanes, luminescent silicone rubbers, azide-alkyne cycloaddition

## Abstract

This study focuses on the synthesis of hybrid luminescent polysiloxanes and silicone rubbers grafted by organometallic rhenium(I) complexes using Cu(I)-catalyzed azido-alkyne cycloaddition (CuAAC). The design of the rhenium(I) complexes includes using a diimine ligand to create an MLCT luminescent center and the introduction of a triple C≡C bond on the periphery of the ligand environment to provide click-reaction capability. Poly(3-azidopropylmethylsiloxane-*co*-dimethylsiloxane) (N_3_-PDMS) was synthesized for incorporation of azide function in polysiloxane chain. [Re(CO)_3_(MeCN)(5-(4-ethynylphenyl)-2,2′-bipyridine)]OTf (Re1) luminescent complex was used to prepare a luminescent copolymer with N_3_-PDMS (Re1-PDMS), while [Re(CO)_3_Cl(5,5′-diethynyl-2,2′-bipyridine)] (Re2) was used as a luminescent cross-linking agent of N_3_-PDMS to obtain luminescent silicone rubber (Re2-PDMS). The examination of photophysical properties of the hybrid polymer materials obtained show that emission profile of Re(I) moiety remains unchanged and metallocenter allows to control the creation of polysiloxane-based materials with specified properties.

## 1. Introduction

Polysiloxanes have unique and useful properties such as high elasticity, transparency, bioinertness, thermal resistance, and gas permeability [1,2,3,4]. These properties determine the wide application of polysiloxanes in biomedicine [5], coatings [6], materials science [7,8], as surfactants [9], or gas separation membranes [10]. Nevertheless, new actively developing applications form new challenges for this class of polymers. In particular, the creation of flexible optoelectronic devices requires not only preserving the above properties, but also adding a fundamentally new one, namely luminescence. Today, luminescent silicone materials show high potential in practical applications and are used in chemical sensors [11], photonics [12], optics [13], biomedicine [14] and optoelectronics [15]. However, despite the potentially broad field of applications, reports of design of luminescent polysiloxanes are still scarce and not systematic, and the number of such polymers represented is still small [16,17,18,19,20,21,22].

Luminescent silicone materials can be made using three main approaches [23]. The first one is to introduce luminophores into the polymer as fillers [21,24]. This approach is the simplest; however, luminescent compounds can be easily extracted from the obtained materials upon contact with organic solvents. In the second approach, luminescent metal complexes are involved in copolymerization reactions with various comonomers [20,21,22]. The approach is perspective; however, some difficulties can appear in high molecular weight copolymers synthesis leading to their low film-forming ability and flexibility. The third method is the polymer modification by luminophores as side substituents of the main chain [16,17,19]. We suggest that the last one opens up new possibilities for the further macromolecular design of silicone materials. In this regard, the synthetic approach of “click” chemistry, namely the copper(I)-catalyzed azido-alkyne cycloaddition (CuAAC), which is widely used in the chemistry of macromolecular compounds and can be utilized to modify copolysiloxane [25]. For example, a copolymer of styrene and N-vinyl carbazole was modified with phosphorescent Ir(III) complexes using this approach [26]. However, data on the use of luminescent metal complexes to modify polysiloxanes are still scarce, and to the best of our knowledge, no reports on the production of luminescent polysiloxanes using the CuAAC reaction have been published to date.

To produce luminescent polysiloxanes using the CuAAC reaction, the emissive metal complex has to be equipped with a terminal triple bond and should also exhibit high stability and predictable chemical behavior. Here, we report mononuclear diimine complexes of rhenium Re(1, 2) that meet these requirements and can act as luminescent centers for functionalized polysiloxanes and as luminescent cross-linking agents for silicone rubbers, respectively. In this work, poly(3-azidopropylmethylsiloxane-*co*-dimethylsiloxane) (N_3_-PDMS) was synthesized and subsequently modified with the [Re(CO)_3_(MeCN)(5-(4-ethynylphenyl)-2,2′-bipyridine)]OTf complex (Re1), as well as the [Re(CO)_3_Cl(5,5′-diethynyl-2,2′-bipyridine)] complex (Re2) acting as a cross-linking agent of N_3_-PDMS. The luminescent properties of the obtained polymers were also investigated.

## 2. Results and Discussion

### 2.1. Synthesis of N_3_-PDMS

**Scheme 1:**
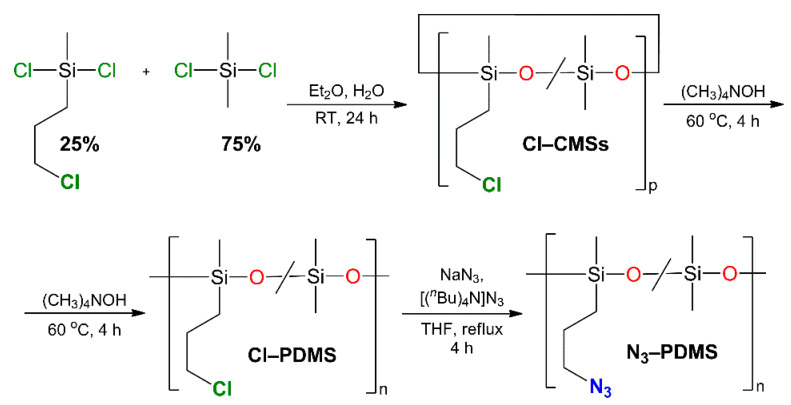
The synthetic route to N_3_-PDMS.

The synthetic procedure included three stages: hydrolysis of initial compounds, polymerization, and azidation. A 24 h cyclo-oligomerization reaction was carried out between dichloro(3-chloropropyl)methylsilane and dichlorodimethylsilane. Next, anionic ring-opening polymerization was carried out, after which the chlorine atoms in the obtained copolysiloxane were replaced by azide groups (Scheme 1).

**Figure 1:**
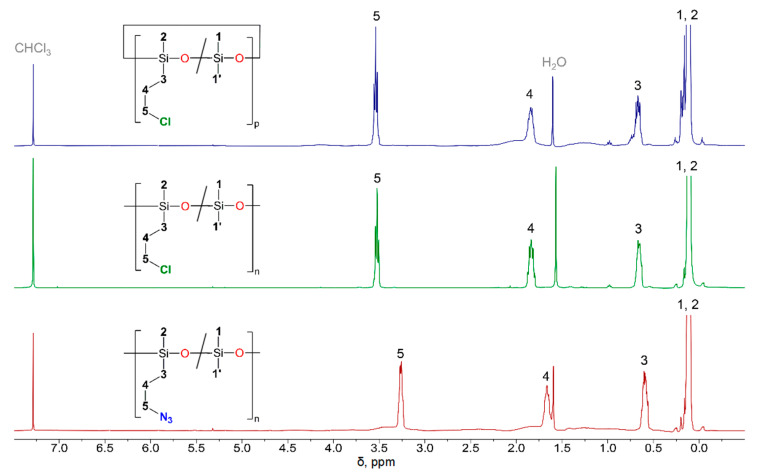
^1^H NMR spectra of Cl-CMSs (blue), Cl-PDMS (green), and N_3_-PDMS (red) with CDCl_3_ as a solvent.

Cyclo-oligo((3-chloropropyl)methylsiloxane-*co*-dimethylsiloxane)s (Cl-CMSs) and poly((3-chloropropyl)methylsiloxane-*co*-dimethylsiloxane) (Cl-PDMS) were synthesized according to the published procedure [27] changing only the ratio of the starting monomers. Dichlorodimethylsilane and dichloro(3-chloropropyl)methylsilane were used in a ratio of 3:1, respectively. It can be found that a decrease of dichloro(3-chloropropyl)methylsilane in the reaction mixture leads to difficulties with the subsequent confirmation of the structure of the obtained polymers due to the low content of the corresponding functional groups. Azidation of the resulting Cl-PDMS was carried out according to a published procedure [28]. The structures of the obtained Cl-CMSs, Cl-PDMS, and N_3_-PDMS were confirmed by NMR spectroscopy (Figure 1 and Figures S1–S3 in Supplementary Materials). The number-average molecular weights of Cl-PDMS and N_3_-PDMS were estimated by gel permeation chromatography (GPC) and amounted to 16,900 g/mol (*Đ* = 1.47) and 14,600 g/mol (*Đ* = 1.50), respectively.

### 2.2. Synthesis of Re1-PDMS

**Scheme 2:**
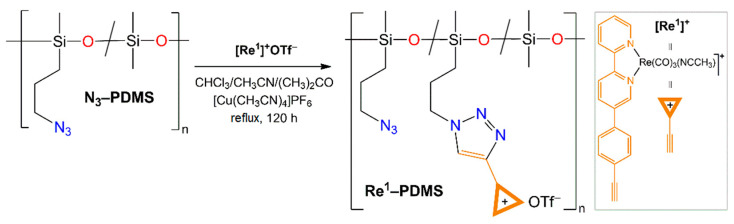
The synthetic route to Re1-PDMS.

The click reaction procedure was optimized on a model system using phenylacetylene (see Supplementary Material section for details, Figures S4–S6), and a copolysiloxane containing Re1 complex was synthesized by CuAAC reaction (Scheme 2). Since the Re1 complex dissolves only in polar solvents (acetone or alcohols) and N_3_-PDMS only in non-polar ones (CHCl_3_, CH_2_Cl_2_, THF), the reaction was carried out in a mixture of solvents: CHCl_3_/CH_3_CN/(CH_3_)_2_CO in a ratio of 3:1:1, respectively.

**Figure 2:**
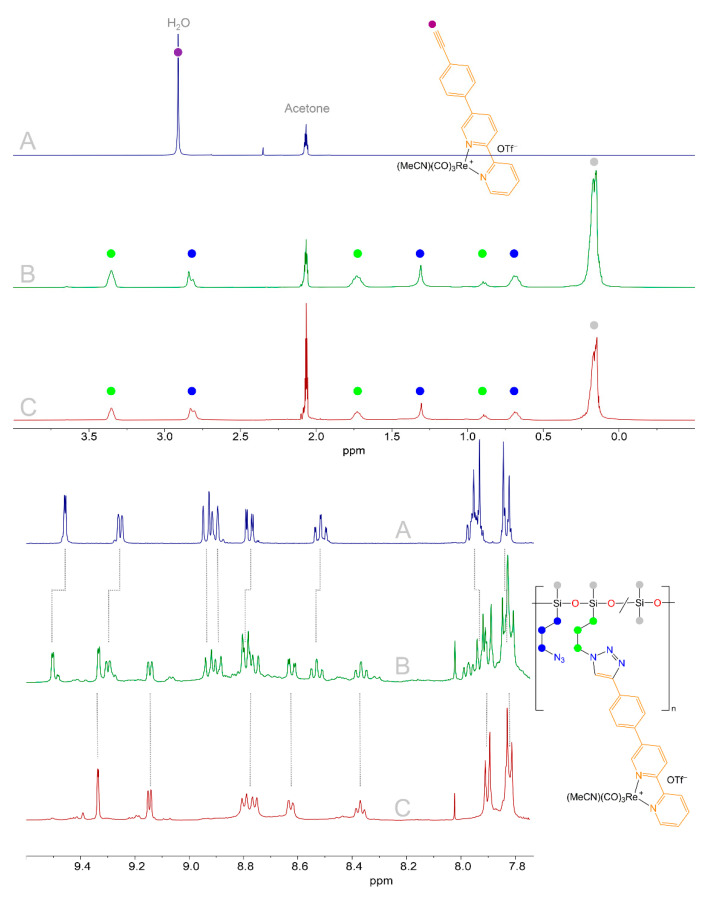
Aliphatic (top) and aromatic (bottom) regions of ^1^H NMR spectra (acetone-d_6_, r.t.): (**A**) the initial Re1 complex; (**B**) reaction mixture after 2-day reaction; (**C**) reaction mixture after 5-day reaction.

The reactions were carried out under reflux within 2 and 5 days. In the case of proceeding within 2 days, the Re1 complex does not fully react and the NMR spectrum of the obtained polymer in the aromatic region contains signals corresponding to the initial complex (δ [ppm] = 7.83; 7.97; 8.02; 8.53; 8.80; 8.90; 8.94; 9.29; 9.50), as well as signals similar in shape and shifted to the high field region (δ [ppm] = 7.89; 8.37; 8.61; 8.64; 8.77; 8.80; 9.14; 9.34). If the reaction proceeds within 5 days, the ^1^H NMR spectrum of Re1-PDMS contains only signals of the product (Figure 2, Figure S7 in Supplementary Material). Thus, according to the proton spectra, all the molecules of the Re1 complex and, accordingly, 20% of the azide groups react (Figure 2 shows aliphatic and aromatic regions of the ^1^H NMR spectra). After the 5-day reaction, solubility of the obtained polymer Re1-PDMS (Scheme 2) in both polar and non-polar solvents also indicated the successful result. A threefold purification of Re1-PDMS was carried out by the reprecipitation from chloroform to acetone and then to CH_3_OH.

**Figure 3:**
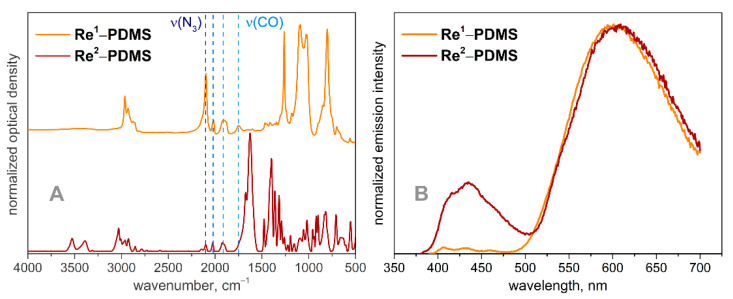
(**A**) FTIR and (**B**) emission spectra (λ_ext_ = 365 nm, r.t.) of Re1-PDMS and Re2-PDMS.

Re1-PDMS was also examined using FTIR spectroscopy. The spectrum contains characteristic absorption bands, which is attributed to the stretching vibrations of unreacted azide groups of the initial polymer N_3_-PDMS and of carbonyl ligands of Re1 complex. The absorption band corresponding to the stretching vibrations of the terminal triple bond C≡C is absent, which can prove that Re1 complex has been involved in the CuAAC reaction completely (Figure 3A).

### 2.3. Synthesis of Re2-PDMS

**Scheme 3:**
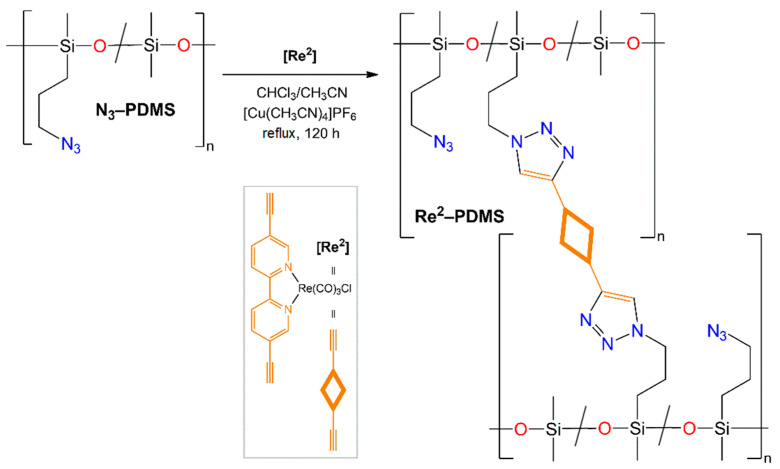
The synthetic route to Re2-PDMS.

The rhenium complex Re2 was used as a cross-linking agent for the synthesis of copolysiloxane Re2-PDMS (Scheme 3). The reaction was carried out in a mixture of solvents CHCl_3_/CH_3_CN in a 4:1 ratio under reflux for 5 days. The product was isolated from the reaction mixture as the white flocculent precipitate and washed with saturated aqueous EDTA-Na_2_ solution, CH_3_CN, and CHCl_3_.

**Figure 4:**
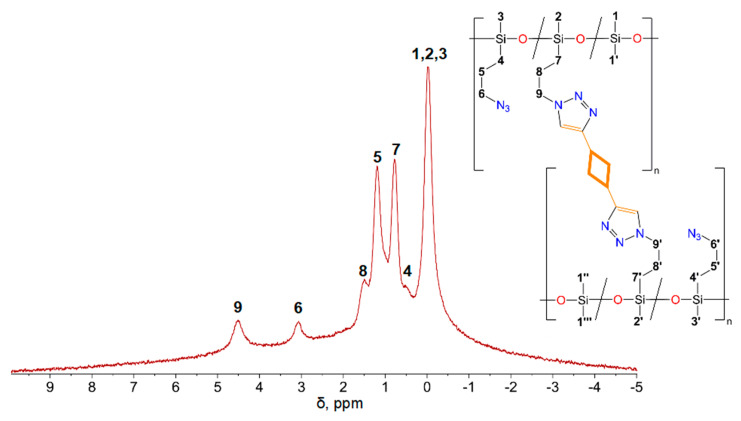
Re2-PDMS solid-state ^1^H NMR spectrum.

The cross-linked polymer Re2-PDMS was investigated by solid-state ^1^H NMR spectroscopy (Figure 4). Although signals corresponding to protons of the rhenium complex Re2 were not observed in the spectrum, the signals in the aliphatic region indicate the successful completion of the reaction.

Examination of Re2-PDMS using FTIR spectroscopy also provide a support for completed reaction and cross-linked polymer formation by CuAAC reaction (Figure 3A).

### 2.4. Re1-PDMS and Re2-PDMS Luminescent Properties

It was found that Re1-PDMS and Re2-PDMS demonstrate photoluminescence in a broad region of the visible spectrum, and their photophysical properties were investigated.

Re1-PDMS and Re2-PDMS are emissive at room temperature with luminescence quantum yields of 0.4% and 0.13%, respectively. Both of the emission patterns are similar and contain two well-separated high energy (HE) and low energy (LE) bands at 406 (λ_00_) and 607 nm, respectively (Figure 3B). The HE band has a well-defined vibrational structure with energy ca. 1400–1500 cm^−1^, which agrees with the stretching frequencies usually observed in the triazole ring [29]. Small values of Stokes shift clearly point to singlet nature of this emission, i.e., fluorescence, and resolved vibronic structure, testifies to the absence of charge transfer and allows to assign HE band to ordinary ππ* intraligand fluorescence of triazole ring [30].

The broad LE band is structureless and exhibits large Stokes shift typical for phosphorescence that, together with their wavelengths and afterglow time in microsecond domain (888.9 ns, Re1 and 901.4 ns, Re2), are compatible with the triplet nature of the emissive excited state ^3^MLCT (M actually represents the {Re(CO)_3_} fragment), which is typical for rhenium(I) tricarbonyl species with diimine ligands [31,32,33,34,35].

The photoemission properties of hybrid “complex-polymer” systems are very similar but not identical (difference in HE band intensity) due to a different amount of triazole rings, on the one hand. On the other hand, the factor of restricted intramolecular motion for Re2-PDMS due to cross-linked structure can play a role in the appearance of difference in photoluminescence of the obtained Re-PDMS systems. It is also important to note that Re1-PDMS and Re2-PDMS are stable under continuous UV irradiation and retain luminescence intensity nearly unchanged, which makes them attractive for potential practical applications.

## 3. Conclusions

Hybrid polysiloxane and silicone rubber were synthesized by a click chemistry approach using the developed procedure of Cu(I)-catalyzed azide-alkyne cycloaddition (CuAAC) between polymer and organometallic Re(I) complexes. The design of Re(I) complexes includes use of a diimine ligand to create an MLCT luminescent center and the introduction of a triple C≡C bond on the periphery of the ligand environment to provide click-reaction capability. Functionalization of copolysiloxane with an organometallic complex Re1 containing terminal C≡C bond at the periphery resulted in the synthesis of a new copolymer Re1-PDMS. The latter is capable of photoluminescence and is suitable for subsequent modification. Using the Re2 complex with two terminal C≡C bonds as a cross-linking agent, a three-dimensional Re2-PDMS polymer network, which can be regarded as a luminescent silicone material, was obtained.

Thus, the developed procedure of CuAAC can be used for preparation of both copolysiloxanes and silicone rubbers functionalized with luminescent backbone substituents. Functionalization and following polymerization or cross-linking do not change the emission properties of the organometallic fragment, which allows to control the creation of polysiloxane-based materials with specified properties.

## 4. Materials and Methods

Dichloro(3-chloropropyl)methylsilane, dichlorodimethylsilane, CuI, 5,5′-dibromo-2,2′-bipyridine, trimetylsilylacetylene, and phenylacetylene were purchased from ABCR GmbH (Karlsruhe, Germany). Tetramethylammonium hydroxide, tetrabutylammonium azide, pentacarbonylrhenium(I) chloride, NaN_3_, Pd(PPh_3_)_2_Cl_2_, CuI, and [Cu(CH_3_CN)_4_]PF_6_ were purchased from Merck KGaA (St. Louis, MO, USA). Sodium ascorbate was purchased from Carl Roth GmbH (Karlsruhe, Germany). Anhydrous Na_2_SO_4_, NaHCO_3_, CuSO_4_·5H_2_O, ethylenediaminetetraacetic acid disodium salt (EDTA-Na_2_), ethyl acetate, chloroform, methanol, diisopropylamine, and acetonitrile were purchased from Vekton (St. Petersburg, Russia). Et_2_O (Vekton, St. Petersburg, Russia), diisopropylamine, and tetrahydrofuran (THF, Vekton, St. Petersburg, Russia) were distilled with sodium/benzophenone system prior to use. Neutral Al_2_O_3_ (Vekton, St. Petersburg, Russia) was activated by heating at 400 °C for 5 h.

5-(4-trimethylsilylethynylphenyl)-2,2′-bipyridine (L1-TMS) was synthesized according to published procedure [36]. 5,5′-trimethylsilylethynyl-2,2′-bipyridine (L2-TMS) was prepared utilizing the adopted protocol described earlier [37].

NMR spectra were recorded on a Bruker Avance III 400 spectrometer at ambient temperature (400 MHz for ^1^H, 100 MHz for ^13^C, respectively). Chemical shifts are given in δ-value [ppm] referenced to the residual signals of non-deuterated solvents: CHCl_3_ (δ = 7.26 (^1^H) and 77.2 (^13^C)) and acetone (δ = 2.05 (^1^H) and 29.8 (^13^C)). High resolution electrospray ionization mass spectra (ESI-HRMS) were obtained on a Bruker microTOF spectrometer equipped with an ESI source. The analyzed samples were dissolved in methanol. The instrument was operated in a positive ion mode using the m/z range of 50–3000. The most intense peak in the isotopic pattern is noted. The estimation of molecular weights was carried out by GPC on a Shimadzu LC-20AD chromatograph equipped with a refractometric detector and a PLgel MIXED-C column (Agilent, Santa Clara, CA, US). Analysis conditions: 40 °C, THF, 1.0 mL/min. The polymer solution in THF (3 g/L) was filtered through membrane filters made of polyvinylidene fluoride, pore diameter 0.22 μm, membrane diameter 13 mm (Macherey–Nagel). The number-average molecular weights (*M_n_*), mass-average molecular weights (*M_w_*), and the degree of dispersion (*Đ = M_w_/M_n_*) were calculated using the Shimadzu LCsolution program using a cubic calibration dependence based on polystyrene standards (*M_n_* = 500–250000). Solid-phase luminescence spectra were recorded on a Fluorog-3 modular spectrofluorimeter (Horiba Jobin Yvon) at ambient temperature. The integration sphere was used to measure the emission quantum yield. Luminescence kinetics were obtained using Time-Correlated Single Photon Counting (TCSPC) method with LED (emission wavelength 265 nm, pulse duration 1 ns) as excitation sources. Infrared spectra were recorded on a Shimadzu IRAffinity-1 FTIR spectrophotometer in KBr pellets (resolution 2 cm^–1^, 40 scans, range 4000–500 cm^–1^).

### 4.1. Synthesis of 5,5′-Trimethylsilylethynyl-2,2′-bipyridine (L2-TMS)

Degassed and filled with argon Schlenk flask was charged with 75 mg Pd(PPh_3_)_2_Cl_2_, 37.5 mg CuI, 375mg 5,5′-dibromo-2,2′-bipyridine, 25 mL of absolute THF, 580 µL trimethylsilylacetylene and 4 mL of dry diisopropylamine. The mixture was stirred at 45 °C for 40 h. After the process finished, the solvent was evaporated. Then the residue was dissolved in 25 mL of dichloromethane and a small amount of activated carbon and water solution containing an excess of NaCN was added. The resulting mixture was filtered through Celites. The organic phase was washed with two portions of water and dried over Na_2_SO_4_. The product was purified by column chromatography (Silica gel, eluted with DCM, R_f_ = 0.35) affording off-white powder, yield 332 mg (79%). ^1^H NMR (400 MHz, (CD_3_)_2_CO): δ 8.74 (d, J = 2.1 Hz, 2H), 8.47 (d, J = 8.3 Hz, 2H), 8.00 (dd, J = 8.2, 2.2 Hz, 2H), 0.30 (s, 18H). ESI HRMS [M + H]^+^ calcd. 349.1551, found 349.1552, [M + Na]^+^ calcd. 371.1370, found 371.1392.

### 4.2. Synthesis of [Re(CO)_3_L1(MeCN)]OTf (Re1)

[Re(CO)_3_Cl(L1-TMS)]. Pentacarbonylrhenium(I) chloride (66 mg, 0.183 mmol) and L1-TMS (60 mg, 0.183 mmol) were suspended in toluene (15 mL) and degassed by purging argon for 15 min upon stirring. Then, the reaction mixture was refluxed for 1.5 h under an argon atmosphere to give an orange solution. The solvent was removed by rotary evaporation and the resulting oil was dissolved in dichloromethane and precipitated in hexane, the solid obtained was washed with pentane and dried. Orange solid, yield 78 mg (85%). The complex was used immediately without future characterization.

[Re(CO)_3_L1(MeCN)]OTf (Re1). [Re(CO)_3_Cl(L1-TMS)] was suspended in 15 mL of acetonitrile and AgOTf (45 mg, 0.173 mmol) was added. The reaction mixture was degassed and refluxed for 12h. The precipitation of AgCl was filtered, the solution was evaporated to give a yellow oil. The crude product was dissolved in dichloromethane and the product was precipitated with diethyl ether. Orange solid, yield 70 mg (66%). ^1^H NMR (400 MHz, Acetone-d_6_): δ 9.46 (d, J = 2.2 Hz, 1H), 9.25 (dd, J = 5.5, 1.4 Hz, 1H), 8.92 (dd, J = 13.1, 8.3 Hz, 2H), 8.78 (dd, J = 8.5, 2.2 Hz, 1H), 8.52 (td, J = 7.9, 1.5 Hz, 1H), 7.98–7.91 (m, 3H), 7.88–7.79 (m, 2H), 2.33 (s, 3H, CH_3_CN^coord^), the signal of proton HC≡C is masked by water signal.

### 4.3. Synthesis of [Re(CO)_3_Cl(L2)] (Re2)

[Re(CO)_3_Cl(L2-TMS)]. Pentacarbonylrhenium(I) chloride (50 mg, 0.14 mmol) and 5,5′-trimethylsilylethynyl-2,2′-bipyridine (49 mg, 0.14 mmol) were suspended in toluene (15mL) and degassed by purging argon for 15 min upon stirring. Then the reaction mixture was refluxed for 3 h under an argon to give a dark orange solution. The solvent was removed by rotary evaporation to give an oil. The product was dissolved in dichloromethane and precipitated in hexane, the solid obtained was washed with pentane and dried in vacuo. Orange solid, yield 78 mg (85%). ^1^H NMR (400 MHz, CDCl_3_): δ 9.08 (dd, J HH= 1.8, 0.8 Hz, 2H), 8.09–8.01 (m, 4H), 0.34 (s, 18H).

[Re(CO)_3_Cl(L2)] (Re2). To a stirred suspension of [Re(CO)_3_Cl(L2-TMS)] (40 mg, 0.06 mmol) in MeOH (15mL) was added K_2_CO_3_ (25 mg, 0.018 mmol). The reaction mixture was stirred for 4 h, then the solvent was removed by rotary evaporation. The product was dissolved in dichloromethane and washed with water. Then, the organic layer was separated and dried over MgSO_4_. The solvent was removed to a minimum by rotary evaporation and the residue was precipitated in hexane. The precipitate was collected, washed with diethyl ether, and dried. Orange solid, yield 40 mg (80%) ^1^H NMR (400 MHz, Acetone-d_6_): δ 9.16 (d, JHH = 1.9 Hz, 2H), 8.76 (d, JHH = 8.5 Hz, 2H), 8.42 (dd, JHH = 8.5, 2.0 Hz, 2H), 4.34 (s, 2H).

### 4.4. Synthesis of Cyclo-oligo(3-chloropropylmethylsiloxane-co-dimethylsiloxane)s (Cl-CMSs)

A total of 23.38 mL of dichloro(3-chloropropyl)methylsilane (*ρ* = 1.204 g/mol, 147 mmol) and 53.2 mL of dichlorodimethylsilane (*ρ* = 1.068 g/mol, 440 mmol) were dissolved in 140 mL of freshly distilled Et_2_O, and then the resulting solution was added dropwise to 220 mL H_2_O at 0 °C. The mixture was stirred at RT for 24 h and extracted with ethyl acetate. The collected organic layer was washed with a saturated aqueous NaHCO_3_ solution, dried over anhydrous Na_2_SO_4_ overnight, and filtered. The solvent was removed under reduced pressure using a rotary evaporator. Colorless liquid, yield 50.56 g (60%). ^1^H NMR (400 MHz, CDCl_3_): δ 3.54 (br. m., 2H), 1.84 (br. m, 2H), 0.67 (br. m, 2H), 0.12 (m, high intensity). ^13^C NMR (100 MHz, CDCl_3_): δ 47.6, 26.7, 14.7, 1.0, 0.7, −0.7.

### 4.5. Synthesis of Poly(3-chloropropylmethylsiloxane-co-dimethylsiloxane) (Cl-PDMS)

In an argon atmosphere, 12 drops of (CH_3_)_4_NOH 25% methanol solution were added to 20 g of Cl-CMSs. The anionic polymerization was carried out in bulk at 60 °C for 4 h in the argon atmosphere. Thereafter, the reaction mixture was heated to 110 °C for 15 min to decompose the initiator residue. The polymer was purified by reprecipitation-decantation 3 times using CHCl_3_ (8 mL) as solvent and CH_3_OH (150 mL) as precipitant. The remaining solvents were removed under reduced pressure (20 mbar, 55 °C) using a rotary evaporator. Colorless viscous liquid, yield 8.08 g (40%). ^1^H NMR (400 MHz, CDCl_3_): δ 3.52 (br. m, 2H), 1.84 (br. m, 2H), 0.65 (br. m, 2H), 0.10 (m, high intensity). ^13^C NMR (100 MHz, CDCl_3_): δ 47.6, 26.7, 15.0, 1.1, 0.7, −0.5. *M_n_* = 16900, *M_w_* = 24800, *Đ* = 1.47.

### 4.6. Synthesis of Poly(3-azidopropylmethylsiloxane-co-dimethylsiloxane) (N_3_-PDMS)

A total of 8 g of Cl-PDMS (22.3 mmol of Cl-incorporating units), 2.2 g of NaN_3_ (33.7 mmol), and 0.3 g of (*n*-butyl)_4_NN_3_ (1.07 mmol) were dissolved in 95 mL of freshly distilled THF. The mixture was refluxed for 24 h in an argon atmosphere. Then another 0.2 g portion of (n-butyl)_4_NN_3_ (0.67 mmol) was added and the mixture was continued to reflux for 24 h in the argon atmosphere. The mixture was filtered, and the solvent was removed under reduced pressure (20 mbar, 55 °C) using a rotary evaporator. The polymer was dissolved in freshly distilled Et_2_O and purified by filtration through activated neutral Al_2_O_3_. Et_2_O was removed by rotary evaporation (20 mbar, 55 °C). Colorless viscous liquid, yield 5.5 g (77%). ^1^H NMR (400 MHz, CDCl_3_): δ 3.27 (br. m, 2H), 1.67 (br. m, 2H), 0.60 (br. m, 2H), 0.10 (m, high intensity). ^13^C NMR (100 MHz, CDCl_3_): δ –0.5, 0.7, 1.0, 14.5, 22.8, 54.1. *M_n_* = 14,600, *M_w_* = 21,900, *Đ* = 1.50.

### 4.7. Synthesis of Copolysiloxane Containing the Re1 Complex (Re1-PDMS)

The synthesis was carried out in a system containing 3 solvents: CHCl_3_, CH_3_CN, and acetone in a ratio of 3:1:1, respectively. The ratio of the Re1 complex to the units containing azide groups was 1:5, respectively.

A total of 10 mg of Re1 complex (0.02 mmol), 32.2 mg of N_3_-PDMS (0.1 mmol of units containing azide groups) and 33 mg of [Cu(CH_3_CN)_4_]PF_6_ (0.1 mmol) were dissolved in 10 mL of a solvent mixture. The mixture was refluxed for 120 h with vigorous stirring in an argon atmosphere. Thereafter, the reaction mixture was washed with a saturated water solution of EDTA-Na_2_. The organic phase was separated, dried over anhydrous Na_2_SO_4_, and filtered. The polymer was dissolved in 2 mL of CHCl_3_, reprecipitated in 15 mL of acetone, and the upper transparent layer of the precipitant was removed using a Pasteur pipette, after which the precipitant was removed under reduced pressure (20 mbar, 55 °C) using a rotary evaporator. The polymer purification process was repeated one more time using CH_3_OH as a precipitant. The precipitant was removed under reduced pressure (20 mbar, 55 °C) using a rotary evaporator. Yellow film, yield: 23.8 mg (56%). NMR data are presented in Supplementary Materials (Figure S7).

### 4.8. Synthesis of Copolysiloxane Containing the Re2 Complex (Re2-PDMS)

The synthesis was carried out in a system containing 2 solvents: CHCl_3_ and CH_3_CN in a ratio of 4:1, respectively. The ratio of complex Re2 to units containing azide groups was 1:10, respectively.

A total of 23.5 mg of complex Re2 (0.04 mmol), 168 mg of N_3_-PDMS (0.4 mmol of units containing azide groups), and 171 mg of [Cu(CH_3_CN)_4_]PF_6_ (0.4 mmol) were dissolved in 10 mL of a solvent mixture. The mixture was refluxed for 120 h with vigorous stirring in an argon atmosphere. At the end of this period, the mother liquor and the formed flocculent precipitate were separated, the precipitate was dried under reduced pressure (20 mbar, 55 °C) using a rotary evaporator. The precipitate was washed with a saturated water solution of EDTA-Na_2_ for 24 h, after which it was separated by decantation and washed with CHCl_3_ for another 24 h. Finally, the precipitate was dried under reduced pressure (20 mbar, 55 °C) using a rotary evaporator.

## Data Availability

All data reported herein are accompanying the present article.

## Supplementary Materials

The following are available online. Description of optimization of the click reaction procedure by a model system with phenylacetylene; ^1^H and ^13^C NMR spectra. Figure S1. ^13^C NMR spectrum of Cl-CMSs; Figure S2. ^13^C NMR spectrum of Cl-PDMS; Figure S3. ^13^C NMR spectrum of N_3_-PDMS; Figure S4. Optimization scheme for the click chemistry reaction; Figure S5. Superimposed ^1^H NMR spectra of the resulting polymers for each of the three reaction systems based on CuSO_4_·5H_2_O/sodium ascorbate (a), [Cu(CH_3_CN)_4_]PF_6_ (b), and CuI (c); Figure S6. ^13^C NMR spectrum of Ph‐PDMS ([Cu(CH3CN)4]PF6 catalytic system); Figure S7. ^13^C NMR spectrum of Re1-PDMS (after 5-day reaction). Table S1. Comparison of the efficiency of catalytic systems.

## Sample Availability

Samples of all the compounds are available from the authors.
