# Supplementary material for: Re(I) Complexes as Backbone Substituents and Cross-Linking Agents for Hybrid Luminescent Polysiloxanes and Silicone Rubbers
Source: Molecules. 2021 Nov 14;26(22):6866. doi: 10.3390/molecules26226866 (PMC8620272; doi:10.3390/molecules26226866)
Supplement: Supplementary file 1 [file molecules-26-06866-s001.zip › molecules-1451799-supplementary.pdf]

Supplementary Material

Re(I) complexes as backbone substituents and cross-linking agents for hybrid luminescent polysiloxanes and silicone rubbers

Egor M. Baranovskii,[1] Victoria V. Khistiaeva,[1,2] Konstantin V. Deriabin,[1] Stanislav K. Petrovskii,[1] Igor O. Koshevoy,[2] Ilya E. Kolesnikov,[3] Elena V. Grachova[1*] and Regina M. Islamova[1*]

[1] Institute of Chemistry, St Petersburg University, 198504 St Petersburg, Russia; baranovskij.1985@mail.ru (E.M.B.); st034645@student.spbu.ru (V.V.K.); deriabin.k@yahoo.com (K.V.D.); s.petrovsky@spbu.ru (S.K.P.)

[2] Department of Chemistry, University of Eastern Finland, 80101 Joensuu, Finland; igor.koshevoy@uef.fi (I.O.K.)

[3] Center for Optical and Laser Materials Research, Research Park of St Petersburg University, 198504 St Petersburg, Russia

* Correspondence: e.grachova@spbu.ru (E.V.G.); r.islamova@spbu.ru (R.M.I.)

Content

NMR spectra of Cl-CMSs, Cl-PDMS, and N_3-PDMS 2

Figure S1. ^{13}C NMR spectrum of Cl-CMSs. 2

Figure S2. ^{13}C NMR spectrum of Cl-PDMS. 2

Figure S3. ^{13}C NMR spectrum of N_3-PDMS. 2

Optimization of the procedure for conducting the CuAAC reaction on a model system 3

Figure S4. Optimization scheme for the click chemistry reaction. 3

Table S1. Comparison of the efficiency of catalytic systems. 3

Figure S5. Superimposed ^{1}H NMR spectra of the resulting polymers for each of the three reaction systems based on $CuSO_4{\cdot}5H_2O$/sodium ascorbate (a), $[Cu(CH_3CN)_4]PF_6$ (b), and CuI (c). 4

Figure S6. ^{13}C NMR spectrum of Ph-PDMS ($[Cu(CH_3CN)_4]PF_6$ catalytic system). 4

^{13}C NMR spectrum of Re1-PDMS 5

Figure S7. ^{13}C NMR spectrum of Re1-PDMS (after 5-day reaction). Resonances 12–25 are assigned to Re moiety. 5

NMR spectra of Cl-CMSs, Cl-PDMS, and N_3-PDMS

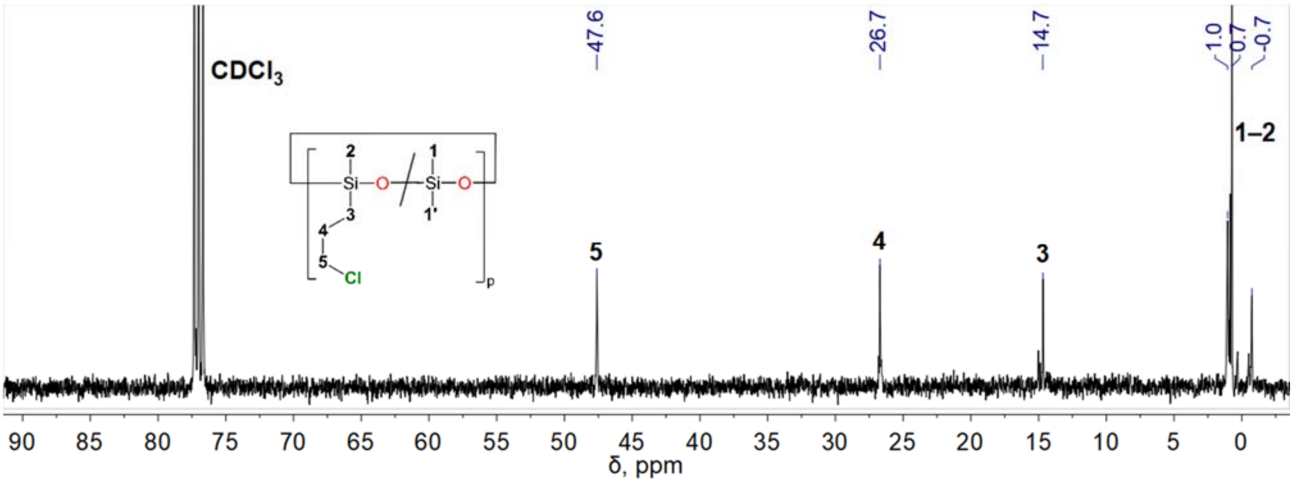

Figure S1. ^{13}C NMR spectrum of Cl-CMSs.

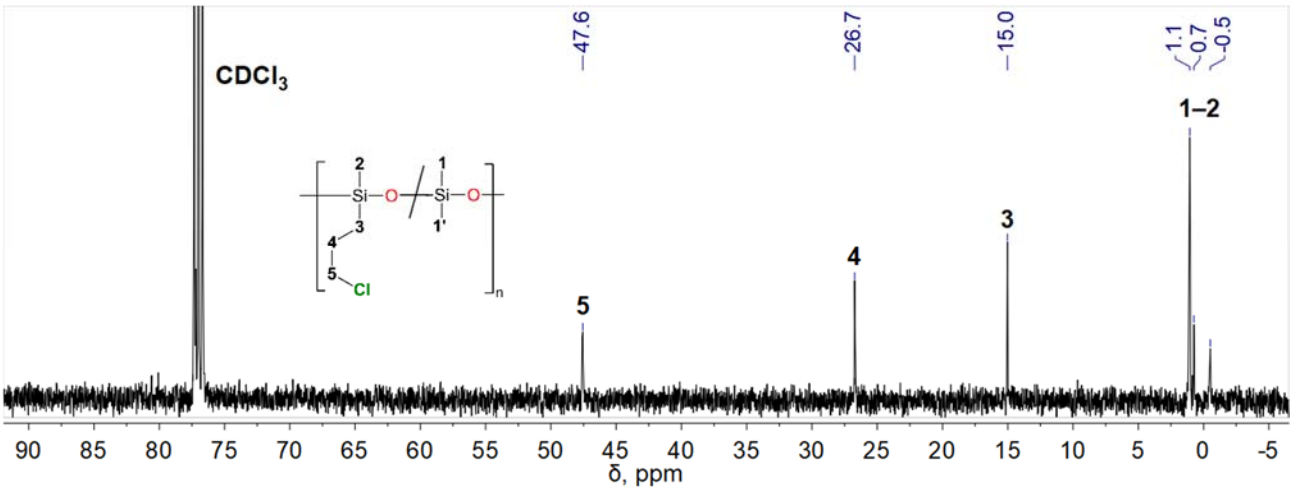

Figure S2. ^{13}C NMR spectrum of Cl-PDMS.

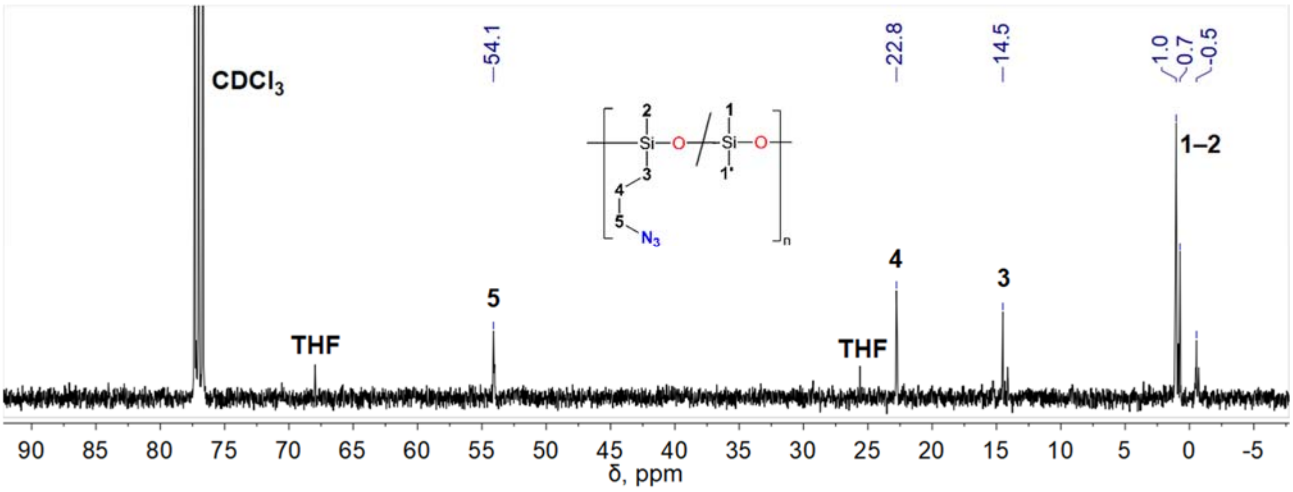

Figure S3. ^{13}C NMR spectrum of N_3-PDMS.

Optimization of the procedure for conducting the CuAAC reaction on a model system

To optimize the procedure, the N_3-PDMS/phenylacetylene system was chosen as a model system. The effect of three different catalytic systems (*i*−*iii*) on the completeness of the reaction was studied (Figure S4).

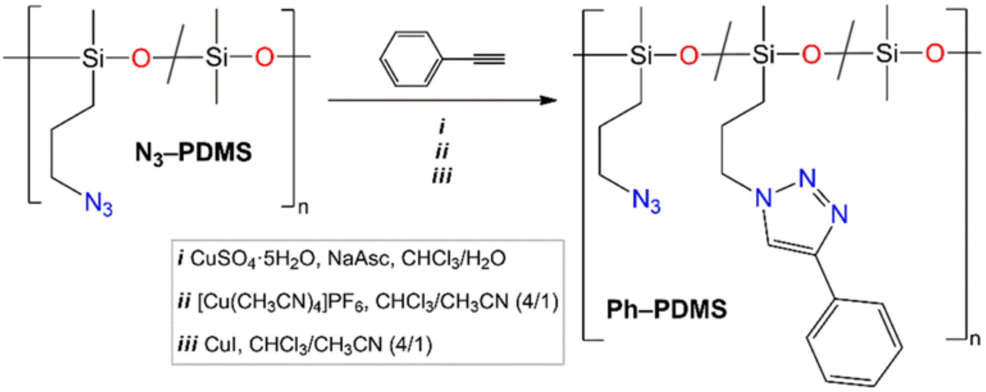

Figure S4. Optimization scheme for the click chemistry reaction.

Three different catalytic systems were used: the $CuSO_4{\cdot}5H_2O$/sodium ascorbate system, as well as $[Cu(CH_3CN)_4]PF_6$ and copper(I) iodide. It was experimentally found that to carry out a click reaction in a model system using $[Cu(CH_3CN)_4]PF_6$ as a catalyst, a mixture of $CHCl_3/CH_3CN$ in a 4:1 ratio, respectively, must be used as a reaction medium. Violation of this ratio leads to a decrease in the solubility of one of the components with the formation of the corresponding precipitate. Based on the results of the ^{1}H NMR spectroscopy analysis (Figure S5), the percentage of reacted azide groups for each system was established from the ratios of the integral intensities of the corresponding peaks (Table S1). $[Cu(CH_3CN)_4]PF_6$ was chosen as the most effective catalyst, which in addition to efficiency allows the reaction to proceed in one phase.

Synthesic method of Ph-PDMS preparation. 350 mg of N_3-PDMS (0.96 mmol of N_3-containing units) and 98 mg (0.96 mmol) of phenylacetylene were dissolved in 30 ml of $CHCl_3$. The resulting solution was mixed with catalyst which was previously dissolved in 7.5 ml of CH_3CN: (*i*) 240 mg (0.96 mmol) of $CuSO_4{\cdot}5H_2O$ and 380 mg (1.92 mmol) of sodium ascorbate dissolved in water, or (*ii*) 358 mg (0.96 mmol) of $[Cu(CH_3CN)_4]PF_6$, or (*iii*) 183 mg of (0.96 mmol) CuI. The mixture was refluxed with vigorous stirring for 24 h. In the case of $CuSO_4{\cdot}5H_2O$/sodium ascorbate system the organic phase was separated, then washed twice with water and filtered off. The resulting solution was washed with a saturated water solution of Trilon B, dried over anhydrous Na_2SO_4, and filtered off. The solvent was removed under reduced pressure (20 mbar, 55 °C) using a rotary evaporator. Yields: 383 mg (86%, $CuSO_4{\cdot}5H_2O$/sodium ascorbate catalytic system), 408 mg (91%, $[Cu(CH_3CN)_4]PF_6$ catalytic system), and 323 mg (72%, CuI catalytic system). NMR data are presented below (Figure S5, Figure S6).

Table S1. Comparison of the efficiency of catalytic systems.

Catalyst	Percentage of reacted N_3-groups
$CuSO_4{\cdot}5H_2O$/sodium ascorbate	55%
$[Cu(CH_3CN)_4]PF_6$	97%
CuI	23%

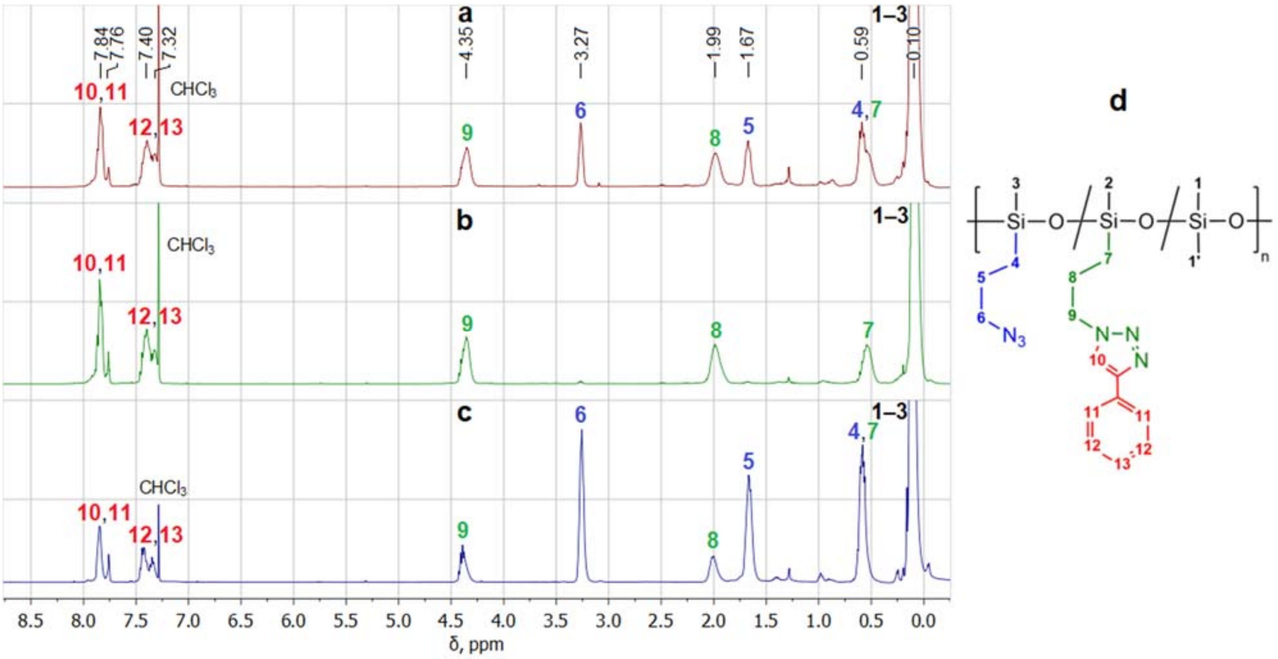

Figure S5. Superimposed ^{1}H NMR spectra of the resulting polymers for each of the three reaction systems based on $CuSO_4{\cdot}5H_2O$/sodium ascorbate (a), $[Cu(CH_3CN)_4]PF_6$ (b), and CuI (c).

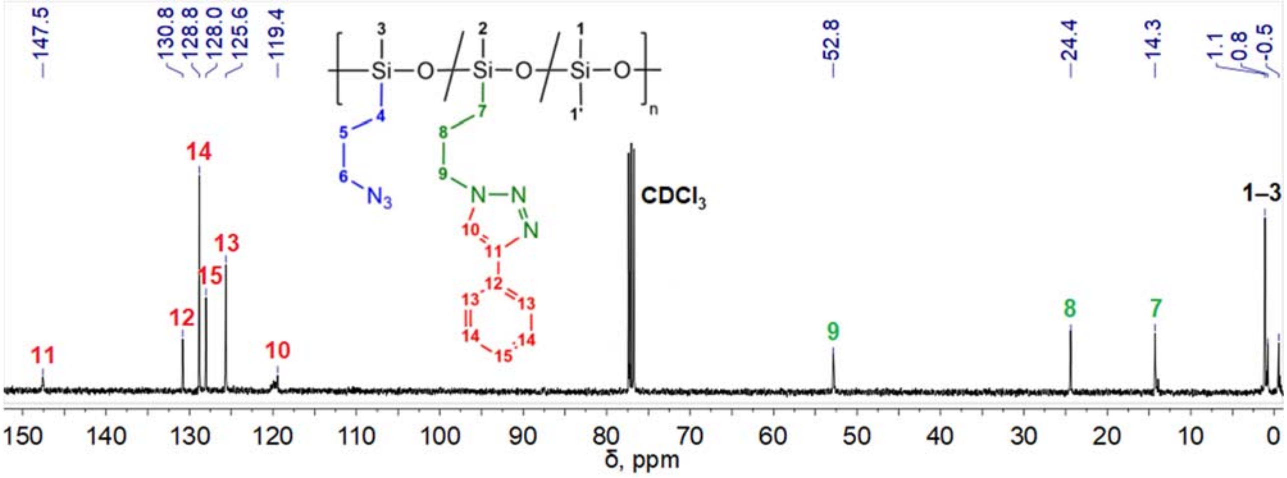

Figure S6. ^{13}C NMR spectrum of Ph-PDMS ($[Cu(CH_3CN)_4]PF_6$ catalytic system).

^{13}C NMR spectrum of Re1-PDMS

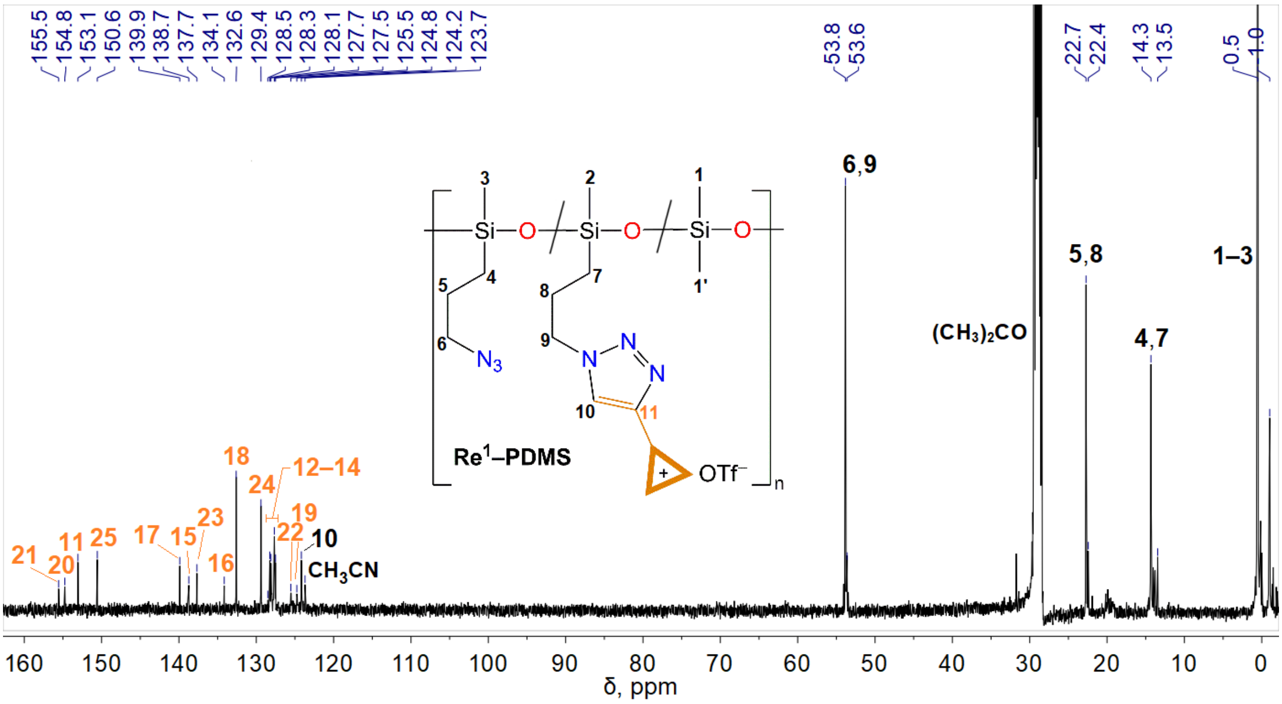

Figure S7. ^{13}C NMR spectrum of Re1-PDMS (after 5-day reaction). Resonances 12–25 are assigned to Re moiety.
